# The Human Gut Microbiota: A Key Mediator of Osteoporosis and Osteogenesis

**DOI:** 10.3390/ijms22179452

**Published:** 2021-08-31

**Authors:** Kevin D. Seely, Cody A. Kotelko, Hannah Douglas, Brandon Bealer, Amanda E. Brooks

**Affiliations:** 1 College of Osteopathic Medicine, Rocky Vista University, Ivins, UT 84738, USA; Cody.kotelko@rvu.edu (C.A.K.); hannah.douglas@rvu.edu (H.D.); Brandon.bealer@rvu.edu (B.B.); abrooks@rvu.edu (A.E.B.); 2 Department of Research and Scholarly Activity, Rocky Vista University, Ivins, UT 84738, USA

**Keywords:** human gut microbiota, microbiome, osteoporosis, osteogenesis, bone health, probiotics

## Abstract

An expanding body of research asserts that the gut microbiota has a role in bone metabolism and the pathogenesis of osteoporosis. This review considers the human gut microbiota composition and its role in osteoclastogenesis and the bone healing process, specifically in the case of osteoporosis. Although the natural physiologic processes of bone healing and the pathogenesis of osteoporosis and bone disease are now relatively well known, recent literature suggests that a healthy microbiome is tied to bone homeostasis. Nevertheless, the mechanism underlying this connection is still somewhat enigmatic. Based on the literature, a relationship between the microbiome, osteoblasts, osteoclasts, and receptor activator of nuclear factor-kappa-Β ligand (RANKL) is contemplated and explored in this review. Studies have proposed various mechanisms of gut microbiome interaction with osteoclastogenesis and bone health, including micro-RNA, insulin-like growth factor 1, and immune system mediation. However, alterations to the gut microbiome secondary to pharmaceutical and surgical interventions cannot be discounted and are discussed in the context of clinical therapeutic consideration. The literature on probiotics and their mechanisms of action is examined in the context of bone healing. The known and hypothesized interactions of common osteoporosis drugs and the human gut microbiome are examined. Since dysbiosis in the gut microbiota can function as a biomarker of bone metabolic activity, it may also be a pharmacological and nutraceutical (i.e., pre- and probiotics) therapeutic target to promote bone homeostasis.

## 1. Introduction

The healing of bone after a fracture is unlike the healing of other organs in the body. Bone is unique in that it heals without the formation of a fibrous scar due to its cellular makeup. After a fracture, a series of processes allow the bone to create a new matrix and structurally and functionally restore the defect. These healing processes are initiated through cell signaling cascades, during which the increased blood flow caused by inflammation allows the infiltration of cells, nutrients, and growth factors to the wound bed, essential elements to begin the healing process [1]. Thus, bone healing can be compromised by conditions that influence and alter natural cell signaling cascades in gut microbiota dysbiosis [2,3]. While microbial dysbiosis is now being recognized as a significant contributor to many human disease states [4,5], its contribution to perturbations in bone remodeling and healing, such as in osteoporosis and osteopenia [6], has only recently been recognized, although it cannot be underestimated.

Part of the impact of microbial dysbiosis on bone healing and ultimately bone health is mediated by the gut microbiota in the trafficking of TNF^+^ T and Th17 inflammatory cells to the bone marrow as well as influencing the overall inflammatory state of the patient, in what is now being called the “brain–gut–bone” axis [7,8,9]. The inflammatory cells recruited to the wound site by a host of growth factors and chemokines begin laying down the extracellular matrix for the new bone, forming a fibrous callus. The callus, which may also be considered woven or immature bone as it develops and matures, is weaker than normal bone but provides a scaffolding for future periosteal ossification on the proximal and distal ends of the fracture [10]. After acute inflammation subsides, mesenchymal stem cells, which have differentiated into osteogenic cells, begin the process of periosteal ossification, forming successively thin layers of bone between underlying healthy bone or cartilage and the fibrous callus, gradually replacing, or strengthening the callus. Importantly, the bone healing process can only occur when there is a balance of osteoclast and osteoblast activity [11].

The balance of osteoclast and osteoblast activity, and hence the bone healing and remodeling processes, are tightly coupled through several signaling pathways, providing a proper balance between resorption and new bone formation [12]. Interestingly, it has also recently been shown that gut microbiota diversity is decreased in osteoporotic patients, leading to a state of dysbiosis [2]. Based on the abundance of metabolite and cell signaling molecules, particularly short-chain fatty acids such as butyrate [13], produced by the gut microbiota, it stands to reason that these states of unbalance may be connected and should be investigated. Furthermore, preclinical animal models have shown that alterations in the gut microbiota can decrease the quality and hence the strength of bone tissue [14], and in germ-free mice (i.e., mice without a gut microbiota), the number of osteoclasts was reduced, leading to increased bone mass [15].

Osteoblasts and osteoclasts are an essential part of bone modeling and healing [16]. Osteoclasts are multinucleated cells that resorb bone and are derived from monocyte/macrophage lineage cells. Osteoclastogenesis is mediated by osteoblasts expressing the membrane-associated cytokine receptor activator of nuclear factor-kappa B ligand (RANKL) [17]. RANKL (receptor activator of NF-kappaB ligand) is a well-known osteoclast inducer. RANKL and co-stimulatory signals govern osteoclastogenesis in the presence of macrophage-colony-stimulating factor (MCSF) [18]. Importantly, osteoprotegerin (OPG) is a competitive antagonist to RANK and will bind to RANK-L, classifying it as an osteoclastogenesis inhibitor [9]. Consequently, resorption of bone and bone remodeling are upregulated by this process. Improper bone remodeling caused by osteoclast defects has been related to various bone disorders, including osteoporosis, rheumatoid arthritis, primary bone malignancy, and skeletal metastases [19].

While the gut–brain axis is now a recognized component of many disease processes, the brain–gut–bone axis is just starting to gain traction, offering potentially new targets and methods to treat bone cellular imbalances that can lead to a primed immune system and subsequently altered bone healing and osteoclastogenesis. This review considers the human gut microbiota composition and its role in osteoclastogenesis and the bone healing process, specifically in the case of osteoporosis. While the body of knowledge pertaining to the microbiota and its effects on various physiological systems is vast, its relationship with bone is only just emerging; however, it is clear, based on both the underlying relationship with osteoclastogenesis as well as the influence of our current pharmaceutical treatments on the gut microbiota, that it needs to be considered as part of a clinical treatment regimen for osteoporosis or post-surgical bone healing.

## 2. Osteoporosis

Osteoporosis, which is primarily considered an unavoidable consequence of aging, is estimated to affect over 200 million individuals worldwide, with an annual healthcare cost of over USD 13.5 billion in the United States alone [20]. This systemic skeletal disease is marked by low bone mineral density (BMD) and structural degradation, which increases the risk of fragility fractures [21]. The concern is warranted as there are an estimated 8.9 million fractures due to this condition annually [20]. The World Health Organization defines osteoporosis as a bone density T-score of 2.5 or more standard deviations (SD) below the average density of healthy young adults with corresponding age and ethnicity. Osteopenia, which is also characterized by decreased bone mass, although not to the same extent as osteoporosis, is defined as a T-score more significant than 1.0 but less than 2.5 SD below the defined average. Imbalanced osteoblasts and osteoclasts are a hallmark of both low BMD and poor bone healing [22].

Osteoporosis is characterized by an increase in osteoclast function, which subsequently increases bone resorption, with a corresponding decrease in bone formation [23]. The most prevalent causes of osteoporosis are menopause and age, as the bone remodeling process is regulated by estrogen, parathyroid hormone, inflammatory cytokines, and vitamin D [24]. Since bone is a plastic substance that undergoes continual remodeling in response to both physiological and extracorporeal factors, increased bone resorption, and concomitant bone loss, regardless of the underlying mechanisms [11,25], presents a complex pathophysiology that can be influenced by genetic predisposition, as well as pharmaceutics (e.g., glucocorticoids), lifestyle, and diet [24,26]. It is this complexity that can make the underlying causes of osteoporosis particularly difficult to isolate and clinically treat; hence, many treatments address the symptomology, doing little to influence the underlying pathology.

## 3. Clinical Treatment of Osteoporosis

While there are a variety of suitable treatments available for osteopenia and osteoporosis, proper clinical management requires a staged approach with careful consideration of the timing of specific interventions. Current treatment recommendations include exercise, supplementation with calcium and vitamin D (main components of ossified bone), and, eventually, bisphosphonate treatment. However, there may be additional ways to treat this condition, which may be more effective and have fewer side effects. In addition, a possible window of opportunity exists between five and ten years after menopause, where bone turnover markers (i.e., c-terminal cross-linked telopeptide (CTX) and Deoxypyridinoline (DPD)) are highest [27].

Many women will develop osteopenia after menopause but do not yet meet the criteria for diagnosis of osteoporosis; this may be the ideal time for initiating a more conservative treatment to prevent the progression from osteopenia to osteoporosis, possibly resulting in better long-term efficacy [3]. Regardless of the timing, clinical treatments can be divided into four primary areas, which vary in their invasiveness: lifestyle modification, nutraceutical supplementation, pharmaceutical intervention, and surgical management. Each of these areas will be explored in the following sections.

### 3.1. Lifestyle Modifications

Lifestyle modifications are indicated uniformly to prevent bone loss in patients at risk for developing osteoporosis. Adequate calcium and vitamin D, exercise, smoking cessation, fall prevention counseling, and avoiding heavy alcohol consumption are all lifestyle strategies that should be included in a comprehensive management strategy, particularly for those with a family history of osteoporosis [24]. Furthermore, individuals in the initial stages of osteoporosis or experiencing osteopenia should avoid using medications that promote bone loss, such as glucocorticoids [28]. Diet modification and exercise are among the two most beneficial and conservative treatment approaches. Studies in rodents show promising results for the addition of high-fiber diets and the resultant increase in the gut microbiome production of short-chain fatty acids (SCFA), particularly butyrate [14], the primary metabolites of microbial fermentation in the gut [29]. SCFAs appear to protect against postmenopausal and inflammatory bone loss, repair intestinal barriers, and prevent the development of osteoporosis in mice [30,31,32]. These results seem to translate to studies in human populations, where an association between increased dietary fiber consumption and bone mineral density has been demonstrated [33,34]. Furthermore, in cases of glucocorticoid-induced bone loss, there is preclinical evidence in a rat model, which found that the addition of dietary kaempferol, a flavonoid found in fruits and vegetables, induced higher expression of osteogenic markers, and functionally seemed to improve callus formation at the site of injury and to reduce bone loss, effectively countering the adverse effects of glucocorticoids on bone health [35].

Alternatively—or, perhaps, in addition—to dietary changes, exercise has demonstrated several beneficial health effects, including improved musculature to support fall prevention and improved bone mineral density (BMD) [26,36]; however, exercise should not be the only treatment for low BMD but a vital component of a comprehensive treatment plan, bearing in mind that not every exercise regimen prescribed is done so with an evidence-based approach and not every patient will be able to perform the ideal exercise plan. Furthermore, it is equally important to remember that although bone is plastic, the benefits of an exercise regimen on bone health require a sustained effort greater than the 3–8-month typical bone remodeling cycle [37]. A comprehensive discussion of the ideal exercise regimen to support bone health is beyond the scope of this review; however, the reader is directed to a good, recent review by Daly et al. on the optimal exercise training program for bone health [37]. Pagnotti et al. also produced a valuable review in which they take a more cellular approach to consider the mechanism of the beneficial effects of exercise [38]. Regardless of the specific mechanism of benefit, the evidence for lifestyle modification indicates that it should be a first-line clinical recommendation to prevent the patient from progressing from osteopenia to osteoporosis.

### 3.2. Nutraceutical Supplementation

Nutraceutical interventions are easily accessible and a relatively inexpensive recommendation that clinicians should consider as first-line measures in the clinical management of osteopenia and osteoporosis. Increased calcium intake supplemented by vitamin D has been shown to reduce the rate of bone mineral loss without harming the intestinal microbiota [39]. Supplemental elemental calcium (usually 500 to 1000 mg/day) should be taken in split doses at meals by patients with insufficient dietary calcium intake, raising their total calcium intake to approximately 1200 mg/day [40]. The total intake of calcium (diet plus supplements) should not routinely exceed 2000 mg/day because of the possibility of adverse effects, including nephrolithiasis, cardiovascular disease, dyspepsia, iron and thyroid hormone dysregulation, and constipation [41]. However, calcium supplementation alone may not significantly decrease fracture risk [42]. There is controversy regarding the dosing of vitamin D. However, it is known that vitamin D3 (Cholecalciferol) promotes calcium uptake in the small intestine [41,43]; therefore, ensuring adequate levels is necessary and may merit supplementation after proper risk and benefit considerations [44]. Prebiotics and probiotics have been found to help with various chronic inflammatory diseases, and there is increasing evidence that they can also help with calcium metabolism and bone health (see Section 4 and Section 5) [45]. Furthermore, the addition of probiotics, antibiotics, or mucus supplementation to mice treated chronically with glucocorticoids prevented the development of glucocorticoid-induced osteoporosis [46].

#### 3.2.1. Probiotic Supplementation

**Figure 1:**
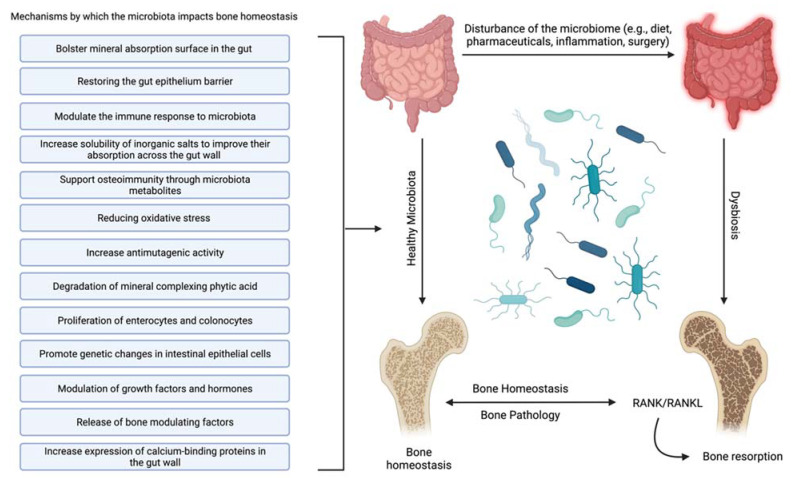
Mechanisms of interaction between the human gut microbiota and bone that lead to increased bone mineral density or suppression of osteoporosis. Created with BioRender.com.

Probiotic supplementation in mice has been shown to increase trabecular bone formation after surgery, indicating that it may be an essential consideration for human bone healing after surgical fracture repair, particularly for osteoporotic patients [47]. This assertion is supported by the fact that supplementation with Bacillus subtilis, lactobacillus, and multispecies probiotic administration have shown beneficial effects not only on the human gut microbiota [48,49] but also on markers of bone turnover [50] and short-term prevention of lumbar spine bone loss [48,51]. Notably, there are conflicting results as to whether probiotics can prevent loss of BMD in the long term [50]. While anecdotal evidence of the benefit of probiotics on bone health is beginning to be supported with rigorous scientific inquiry [52,53], a complete picture of the various proposed mechanisms of action supports the evidence. The interaction between lactose-based prebiotics and probiotics reduces osteoporosis through multiple mechanisms [52,54,55]. The following have been identified as mechanisms underlying the interaction of a healthy gut microbiota that leads to increased bone mineral density (Figure 1).

#### 3.2.2. Mechanisms of Microbiota Impacts

- Increase solubility of inorganic salts to improve their absorption across the gut wall [56]—The availability of inorganic salts such as phosphate is critical for bone mineral deposition by osteoblasts and bone homeostasis [57]. This effect may be partially due to the metabolism of mineral complexed phytic acid [58] by microbial synthesized phytase into inorganic phosphate and a myoinositol phosphate derivative [52].
- Bolster mineral absorption surface in the gut—By promoting the proliferation of enterocytes and colonocytes, gut microbiota homeostasis is mediated, and mineral absorption in the gut is supported. Furthermore, increased colonocyte metabolism has been documented to promote obligate anaerobes, which are known to metabolize fiber, thereby increasing SCFAs, which is critical for bone homeostasis, among other physiological functions [59].
- Restore and maintain gut epithelium barrier—Enhancing the gut’s barrier function is integral to gastrointestinal immunity [60]. A healthy gut epithelial barrier prevents the hyperpermeability that comes with damaged tight junctions [61]. Hyperpermeability or a “leaky gut” leaches unusually high levels of inflammatory cytokines, producing systemic inflammation and leading to hyperactive osteoclasts and bone degradation [62].
- Support osteoimmunity through microbiota metabolites—Short-chain fatty acids (SCFAs) produced by the gut microbiota offer anti-inflammatory effects by inhibiting the activation of nuclear factor kappa-light-chain-enhancer of activated B cells, reducing auto-immune inflammation [63]. Additionally, SCFAs, specifically propionate and butyrate, metabolically reprogram osteoclasts by downregulating TRAF6 and NFATc1 to inhibit osteoclastogenesis and bone resorption, effectively increasing bone density without directly altering osteoblasts [30].
- Reduce oxidative stress [64]—Oxidative stress is documented to cause excessive osteocyte apoptosis, which generates an imbalance favoring osteoclastogenesis, leading to increased bone remodeling, turnover, and loss [65]. Strain-specific probiotics can relieve oxidative stress by producing several antioxidant molecules (e.g., glutathione, folate, and exopolysaccharide). In addition, the SCFAs produced by several gut microbiota can also help to relieve oxidative stress by promoting the production of antioxidant molecules [66,67].
- Modulate the immune response to microbiota [68]—The effects of normal gut microbiota are appreciated when discussing the abnormal rather than the normal. In an abnormal state, the immune system’s reaction to microbiota stimulation leads to an increase in circulating osteoclastogenic cytokines through the action of T-cells. This degradative process is not active in normal gut microbiota states [8].
- Promote genetic changes in intestinal epithelial cells [69]—Although it is not completely clear how they accomplish it, specific gut microbiota can prompt the genetic modification of cells. Recently, it was shown that Bifidobacterium lactis species upregulated cyclooxygenase-1 (Cox-1) and downregulated Cox-2 gene expression in a Caco-2 cell culture model. This outcome is thought to lead to a decrease in tissue damage and inflammation [70].
- Increase antimutagenic activity [52,71,72]—Although this capability has largely been explored in the context of cancer [73], certain species of lactic acid microbiota can bind potent mutagens such as pyrolyzates [74,75] and heterocyclic amines [76,77] in the gut to decrease the mutagenic activity of these compounds. Reducing DNA damage reduces inflammation, protects the gut wall, increases mineral absorption, and suppresses osteoporosis [78].
- Increase expression of calcium-binding proteins in the gut wall—Increasing calbindin-D9k gene expression in the gut wall can increase the ability to absorb calcium [79], effectively suppressing bone degradation and promoting bone deposition by suppressing the actions of parathyroid hormone [39]. In addition, enhanced calcium absorption and inhibition of parathyroid hormone activity and insulin-like growth factor 1 production can also modify the development of osteoclasts and osteoblasts [80,81].
- Modulation of growth factors and hormones [82]—The gut microbiota should be considered an endocrine organ based on the plethora of secreted molecules. Specifically, the gut microbiota promotes the production of IGF-1 through a proposed SCFA-mediated pathway [83]. IGF-1 is known to stimulate the differentiation of osteoblasts, osteoclasts, and chondrocytes. The gut microbiota may also enhance bone degradation through a cortisol-mediated interaction [82,84]; however, the evidence is indirect, and the precise mechanism is unclear. The gut microbiota also modulates gut serotonin production, a molecule that interacts with bone cells and has been suggested to act as a bone mass regulator [85].

### 3.3. Pharmaceuticals

Patients who are at high risk of fracture or who have experienced a fragility fracture are most likely to benefit from a pharmaceutical adjunct therapy in addition to lifestyle and other more conservative interventions. Therefore, it is preferable to design a patient’s treatment strategy based on their fracture risk, which is assessed by a combination of bone mineral density (BMD) and clinical risk factors [86]. Notably, although there are many pharmaceutical options to support bone balance and remodeling, their success is often dictated by patient compliance with most pharmaceutical treatments. Hence, the clinical use of such interventions must include patient counseling. Additionally, before initiating pharmacologic therapy, all patients should have within-range blood calcium and 25-hydroxyvitamin D levels, and nutraceutical intervention should be given if food consumption is insufficient. Pharmacological therapies for osteoporosis can be subclassified as either antiresorptive, anabolic, or hormone modulating agents (Table 1).

Bisphosphonates, a class of antiresorptive drugs, have been widely used since their first use in bone disorders was described in the 1960s. Progressive new frontline treatments are rising in popularity, though, such as denosumab and anabolic agents including parathyroid hormone-related peptide analogs and anti-sclerostin antibodies [87,88]. A complete description of specific members of this drug class with their benefits and side effects is beyond the scope of this review; however, other good reviews consider these topics and the clinical implementation of this class [89]. In addition, patients who have recently had a fragility fracture, such as a hip fracture, should consider therapeutic pharmacological treatment for osteoporosis due to their increased risk of a second fracture [90].

### 3.4. Fracture in Osteoporotic Patients: Surgical Fracture Reduction

Osteoporotic patients have poor bone quality, decreased bone mineral density, and reduced potential for bone regeneration [91], placing them at higher risk of fragility fractures. Altered bone mineral properties increase risk of implant failure after open reduction and internal fixation (ORIF) or total joint arthroplasty due to fixation insufficiency [92]. Compared to healthy individuals, those with osteoporosis form a weaker callous and bony union, contributing to the aseptic loosening of the implant [92,93]. The use of bisphosphonates in conjunction with surgical repair may improve the formation of the callous [94,95,96]. Revision surgeries and periprosthetic fractures are common in total knee and total shoulder arthroplasties in individuals with osteoporosis [97,98]. In spinal fusion patients, pre-existing osteoporosis leads to extended hospital stays, increased medical costs, and higher rates of revision surgery within the first two years [99,100].

Surgeons performing these operations should be aware of the osteoporotic status and recognize the possibility that augmentation of the procedure with lifestyle modifications, supplements, or pharmacological treatments may be necessary [90,101]. However, it is unclear whether the treatment choice is or should be modified based on the underlying mechanism of bone remodeling imbalance. Recently, a multistakeholder coalition was assembled by the American Society for Bone and Mineral Research to develop evidence-based treatment recommendations. The reader is referred to the 2020 published recommendations to prevent second fractures [90]. Unfortunately, these recommendations did not consider the impact of the gut microbiota on bone remodeling.

**Table 1:** Mechanisms of action and treatment recommendations for osteoporosis.

Intervention	Mechanism	Treatment Recommendation	References
Lifestyle Modification			
Exercise	Exercise-induced mechanical loading increases bone mass by regulating the hormones, cytokines, signaling pathways, and noncoding RNAs in bone metabolism.	Patients who have osteopenia or osteoporosis, or would like to prevent it, should exercise for at least 30 min three times a week. Resistance training, jogging, jumping, and walking are generally considered effective.	[102,103,104,105]
Diet	1. Reactive oxygen species induce the apoptosis of osteoblasts and osteocytes. Excessive osteocyte apoptosis is linked to oxidative stress, which causes an imbalance in favor of osteoclastogenesis, resulting in more significant bone remodeling turnover and loss.	1. A diet high in antioxidant-rich foods, such as foods high in polyphenols, supports antiresorptive therapies for the treatment and prevention of bone loss.	[62,65,106,107,108,109]
	2. In chronic inflammatory states, overproduction of cytokines such as tumor necrosis factor, interleukin-1 (IL-1), IL-6, and IL-17 is linked to inflammation. In addition, specific cytokines can impede osteoblast function; their overexpression during inflammation leads to excessive bone degradation, primarily due to hyperactivation of osteoclasts.	2. An anti-inflammatory diet may delay the course of osteoporosis by regulating inflammatory activity, modifying the lipid profile, boosting antioxidant levels, and altering the gut’s microbiota.	
Smoking Cessation	1. Smoking lowers circulating estrogen levels, as seen by higher blood concentrations of follicle-stimulating hormone and luteinizing hormone. Smoking may also increase bone resorption, causing a rise in blood calcium levels, a drop in serum parathyroid hormone levels, and increased urine hydroxyproline and pyridinoline excretion.	Smoking cessation is strongly recommended to all patients concerned with their skeletal health.	[110,111,112]
	2. The chemical composition of smoking (and vaping to a lesser extent) can interfere with other therapeutic measures included in a comprehensive treatment regimen.		
Nutraceuticals			
Calcium with Vitamin D	Vitamin D3 (Cholecalciferol) increases calcium uptake in the small intestine. Increased calcium in the blood promotes bone deposition and suppresses bone resorption.	Total calcium intake (diet + supplements) should approximate 1200 mg/day. Women should also ingest a total of 800 international units of vitamin D3 daily.	[113,114,115]
Probiotics	Can improve calcium balance, prevent secondary hyperparathyroidism, and attenuate age-related increase in bone resorption and bone loss via multiple proposed mechanisms outlined in Section 3 of this review.	No probiotic strategy is currently included in the standard of care or as a primary treatment for osteoporosis.	[52,53,116,117]
		It may benefit overall bone health if consumed daily as preparations with active live cultures containing bacteria, such as *lactobacilli*, *lactococci*, or *bifidobacteria* isolated from natural environments.	
Pharmaceuticals: Antiresorptive Agents			
Bisphosphonates	Nitrogen-containing bisphosphonates selectively inhibit farnesyl pyrophosphate synthase (FPPS) within osteoclasts, inhibiting osteoclast activity.	Oral bisphosphonates are considered as first-line treatment. However, alendronate or risedronate are commonly used and are considered safe and efficacious.	[95,118,119]
Denosumab	Binds with high specificity and affinity to the cytokine RANKL, thereby inhibiting its action; as a result, osteoclast recruitment, maturation, and action are suppressed, and bone resorption slows.	Used as initial therapy in certain patients at high risk for fracture, such as older patients who have difficulty with the dosing requirements of oral bisphosphonates or who have markedly impaired renal function.	[120,121,122]
Pharmaceuticals: Anabolic Agents			
PTH/PTH-RP Analogs	Stimulates bone formation and activates bone remodeling through RANK/RANKL. Intermittent administration of recombinant human PTH or PTHrP has been shown to stimulate bone formation more than resorption.	Indicated in severe cases of osteoporosis (T-score of ≤−3.5 even in the absence of fractures, or T-score of ≤−2.5 plus a fragility fracture). May prescribe for patients with osteoporosis who cannot tolerate bisphosphonates or who have contraindications to oral bisphosphonates and patients who fail other osteoporosis therapies.	[123,124,125]
Romosozumab	Sclerostin inhibits WNT/catenin signaling in osteoblasts and osteocytes, decreasing OPG expression. Direct actions of sclerostin on osteocytes stimulate RANKL expression. Monoclonal anti-sclerostin antibody (Romosozumab) inhibits sclerostin and enhances osteoblast function to improve bone mass and reduce fractures.	Not considered initial therapy for most patients with osteoporosis. Candidates include patients with multiple fragility fractures, those at high risk for fracture, or those who cannot tolerate any other osteoporosis therapies.	[88,126,127]
Pharmaceuticals: Hormone Modulators			
Selective Estrogen Receptor Modulators	Acts upon estrogen-sensitive tissues and functions as an estrogen agonist in bone to prevent bone loss and has estrogen antagonist activity to block some estrogen effects in the breast and uterine tissues. Decreases bone resorption, increasing BMD and decreasing fracture incidence.	Usually recommended for osteoporosis when there is an independent need for breast cancer prophylaxis.	[128,129]
Estrogen/Progestin Therapy	Estrogens regulate the activity of bone-forming osteoblasts and bone-resorbing osteoclasts, which regulate bone mass and strength. The cellular actions of estrogen are mediated primarily through estrogen receptor alpha (ERα), which is present in estrogen-sensitive tissues such as bone.	No longer a first-line approach. Indicated for postmenopausal women with persistent postmenopausal symptoms and an indication for antiresorptive therapy who cannot tolerate other drugs.	[130,131,132]

## 4. Human Gut Microbiota: Physiologic Role

Over 90% of cells in the human body are microbes, most of which are located in the distal gut [16]. While isolating and growing each particular species is seemingly insurmountable and often impossible due to the anaerobic and other physiologic conditions of the gut, metagenomics and biostatistical inference analyses such as PICRUSt [133] have allowed the identification of a human gut microbial profile considering both the composition and function of the physiologic human gut microbiota. In addition to the 553 bacterial species previously known to inhabit the gut, metagenomic sequencing research revealed 1952 unclassified bacterium species in the human gut microbiome [5,134]. While the mechanistic understanding of the microbiome’s effect on specific physiologic processes is still in its infancy, the gut microbiome, directly and indirectly, regulates human nutrition, metabolism, vitamin production, immune system function, and molecular and cellular translocation across the gut endothelium barrier [135,136]. While a comprehensive discussion on the known composition of the gut microbiota is beyond the scope of this review, Almeida et al. provide an excellent, detailed discussion on the characteristic typography of gut microbiota relative to their location for the reader’s further inquiry [134].

The importance of mutualistic, gut-bacterial communities in human health has been underlined by accumulating research over the last two decades. The pathogenesis of chronic, non-communicable, clinical conditions linked to alteration in the gut microbiome or dysbiosis of the gut microbiota includes inflammatory bowel disease [137], obesity [138], metabolic disease [139], malnutrition [140], neurological disorders [141], malignancy [142], and cardiovascular disease [143]. Colonocyte metabolism appears to control the transition between homeostatic and dysbiotic microbiota communities. Colonocyte metabolism involves oxidative phosphorylation during homeostasis, resulting in increased epithelial oxygen demand. The resulting epithelium hypoxia aids the maintenance of a microbial population dominated by obligate anaerobic bacteria, which assist the host by turning fiber into fermentation products that may be ingested. Conditions that change the colonic epithelial metabolism enhance epithelial oxygenation, leading to a rise in facultative anaerobic bacteria, a sign of dysbiosis in the colon [59]. Hence, it is becoming abundantly clear that the gut microbiota composition and relative populations of bacterial species associated with health along various physiological axes must be considered and protected from treatment-induced dysbiosis, either indirectly through colonocytes or directly acting on the gut microbiota whenever possible. This elucidation poses a particularly vexing problem as the nature of the nexus between human health and gut microbiota, the relative abundance of distinct species, and their essential functions in various physiological systems pose a highly complex network of interactions that should be of significant consideration in biomedical research.

This network of interactions that can be affected by gut microbiota dysbiosis extends beyond those directly connected to the gut. There is mounting evidence that the microbiome can play a significant role in the health and function of organs outside of the gut. Disruption of the microbiota balance, and thus the integrity of intestinal tissues, results in increased translocation of microbiota and their bacterial byproducts (e.g., lipopolysaccharides, microbial-associated molecular patterns (MAMPs), and SCFAs), evoking both systemic and local immune responses [144], imparting the ability of the gut microbiota to impact distant organs such as bone [4]. Of particular concern for bone health is the gut microbiota’s fermentation of dietary fiber to yield short-chain fatty acids (SCFAs), endogenous signals that play significant roles in lipid homeostasis and inflammation reduction [145]. Studies have also suggested that increased SCFAs positively impact BMD [30,31]. Thus, SCFA absorption is a nutritional consideration for patients and could have implications for the overall nutrition status of a patient, ultimately affecting physiological processes such as healing [146].

### Human Gut Microbiota: Role in Osteoporosis and Osteoclastogenesis

Discovering that the host’s microbiome can influence even remote tissues has given rise to the field of osteomicrobiology, the study of microbiota in bone health, and the processes through which the microbiota influences skeletal growth, bone aging, and pathologic bone loss. The human gut microbiota and the host interact positively, impacting a dynamic equilibrium that influences bone mass [147]. Recent studies in osteomicrobiology have linked changes in bone phenotype to changes in gut flora. A balanced, “healthy” microbiome appears to be necessary to prevent sex hormone deficiency from inducing bone loss, a supposition supported by evidence that the supplementation of probiotics in the diets of ovariectomized mice led to the reversal of the pathogenic process of osteoporosis [8,47]. Furthermore, an examination of several studies that have evaluated the amount and diversity of bacterial populations in the gut of patients with osteoporosis has made it clear that osteoporotic adults appear to have reduced diversity of organisms, with increases in certain species such as *Fusobacterium*, *Dialister*, *Faecalibacterium*, and *Tolumonas* and decreases in *Bacteroides* and *Roseburia* spp. [2,3,48,148]. Initial conjecture about the underlying mechanism of such an effect leads to a potential immune-mediated effect. Indeed, part of the association between the gut microbiota and bone healing and remodeling is moderated by immune cells.

Many cell signaling cascades necessary for immune cell maturation and function, in part, depend on the gut microbiota for continued optimal function and homeostasis throughout life. Though incredibly complex, the leading hypothesis of recent literature regarding the connection between overall bone health and the gut microbiome centers around T and Th17 immune cells [7,8]. In a T-cell-dependent mechanism, the immune system’s reaction to microbiota stimulation increases circulating osteoclastogenic cytokines [8]. Furthermore, although the role is not entirely understood, it appears that Th17 immune cells are an integral member of the osteoclast population; thus, it can be postulated that any imbalance of the gut microbiome may, in turn, affect a critical immune mechanism necessary for bone homeostasis. Beyond proposed T-cell-mediated interactions, the broader immune system’s reaction to the microbiome results in the creation of a number of circulating cytokines and cell-based immune effectors that have a significant impact on bone cells, indicating that therapeutic approaches to treat bone remodeling and bone healing disorders may benefit from considering the health of the gut microbiota. However, these limited studies clearly show that a complete picture of the impact of microbial imbalance in the development of osteoporosis is critical; illuminating this relationship in the context of bone healing and osteoclastogenesis is fundamental to clinical implementation.

Although the influence of the gut microbiota on the host immune system has a comparatively long, documented history, it is not the only potential mechanism by which the gut microbiota may influence bone health. Steroid hormones, parathyroid hormone (PTH), and vitamin D metabolites may all be affected by the microbiota [13]. Furthermore, bacteria-derived compounds, such as vitamins, may reach the bloodstream and directly impact bone cell activity [53,136]. Alternatively, the gut microbiota might influence the development of osteoporosis through its effect on host micro-RNAs (miRNAs), which are non-coding RNA molecules involved in the regulation of gene expression. Several miRNAs can influence transcripts connected to osteoblast differentiation in osteoporosis, such as miRNA-33-5p [149], miRNA-194 [150], and miRNA-433-3p [151]. Although it is not fully understood, in a feedback mechanism that supports microbial dysbiosis of the gut, microbiota may influence host miRNAs, which in turn may suppress osteoblast development, posing a barrier to osteoporosis repair [152]. Of particular clinical interest may be the recent suggestion that miRNA and probiotics may play an essential role in host–microbe intercommunication [153]. Beyond the effects on miRNA, Uchida et al. also considered a transcriptome-level analysis, finding transcripts for both anabolism and catabolism to be upregulated in the presence of commensal microbiota [154].

While nucleic acid-based influences continue to be revealed, microbial-produced metabolites and growth factors may have a more direct effect. Insulin-like growth factor 1 (IGF-1), a growth factor known to impact bone via endocrine and paracrine–autocrine mechanisms, also needs to be considered as a possible pathway for microbial impacts on bone [80]. IGF-1 is a potent bone regulator. Exogenous IGF-1 increases longitudinal femur development, and cartilage-specific deletion of the receptor reveals that IGF-1 is required for growth plate maturation and secondary ossification center development. Mice with a defined microbiota had greater IGF-1 levels than germ-free (GF) mice [155]. However, it is unclear if colonizing adult GF mice with microbiota is enough to raise IGF-1 levels or if IGF-1 levels stay increased in older colonized mice. At 1 and 8 months after colonization, colonized mice had substantially higher IGF-1 levels than GF controls, and a tendency toward higher serum IGF-1 was seen as early as seven days following colonization. Additionally, although colonization with gut bacteria may promote local IGF-1 synthesis, the effect on circulating IGF-1 was not as substantial. In a somewhat parallel experiment, drosophila insulin/IGF-like peptide activity was raised in sterilized flies colonized with healthy microbiota, and IGF-1 was shown to mediate the microbiome’s influence on postnatal growth. The evidence is now clear; the gut microbiota can influence the levels of IGF-1, and IGF-1 has a significant impact on bone development and healthy maintenance [156].

Overall, there are multiple potential pathways by which the gut microbiota can impact bone healing and remodeling through osteoclastogenesis (see Section 3.2). The abundance of direct, indirect, and observational evidence of such an effect is overwhelming. It potentially necessitates revised clinical guidelines that protect, support, and perhaps even restore the health of the gut microbiota while simultaneously promoting a healthy bone metabolism, particularly during conditions of cellular imbalance (i.e., osteopenia and osteoporosis).

## 5. Bioactive Compounds and the Human Gut Microbiome

**Figure 2:**
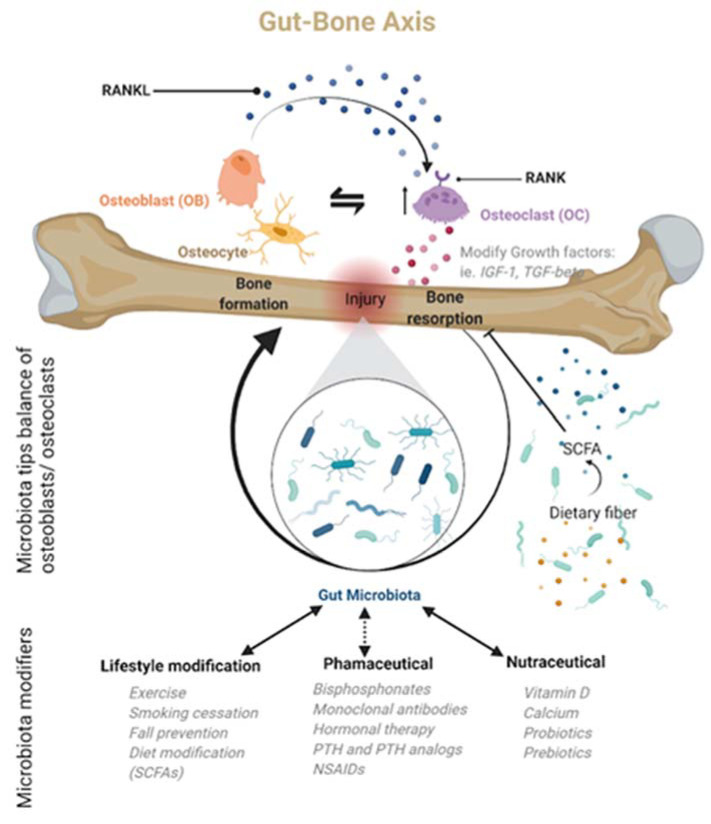
The gut microbiota is only now being recognized as a central regulator of bone homeostasis, particularly after an injury, in what is now being called the gut–bone axis. Many of the regulatory actions are mediated by the microbiota’s metabolic products. Based on this newly recognized microbiota role, bone homeostasis or dysbiosis may be modulated by lifestyle changes, nutraceuticals, or pharmacological agents. Created with BioRender.com.

While it is now clear that a working knowledge of how the microbiota affects bone health naturally through a variety of immune regulators, cytokines, miRNAs, hormones, and other small molecules is essential for clinical orthopedics, it is equally clear that a variety of interventional bioactive compounds (antibiotics, prebiotics, and probiotics) can have a profound impact on the growth, development, and function of the gut microbiota. Bioactive compounds can be both beneficial and harmful in the overall function of the gut microbiota, often altering the balance of species present, potentially leading to dysbiosis. Unfortunately, imbalances early in life can lead to significant morbidities later in life. However, more research is needed to elucidate how this long-term sequence occurs, particularly in osteoporotic patients. Alternatively, in adult patients, emerging research supports the microbiome’s connection to overall health by solidifying the benefits of a healthy diet, with prebiotics and probiotics, in conjunction with antibiotic stewardship. Indeed, there is a growing body of evidence that the optimization of prebiotics, probiotics, and dietary fibers has reproducible health benefits, with specifically lasting benefits on bone health, including decreased incidence of postmenopausal osteoporosis in women [45], keeping in mind that the clinical recommendations for such nutraceuticals remain enigmatic at best due to a lack of regulation [157]. The above notwithstanding, a broad array of research supports the aforementioned connection. McCabe et al. demonstrated that probiotics benefit bone growth, density, and structure when given during conditions of dysbiosis [158]. Echoing this connection, Zmora further mentions that there can be bacterial colonization resistance to standard probiotics, lending the idea that bioactive drugs such as prebiotics, probiotics, or antibiotics will need to take on an unprecedented, personalized treatment approach in the future [159]. Indeed, the impact of these and other exogenous bioactive compounds on the gut microbiota can vary based on the patient’s age and supports the need for a more personalized approach. With newer technologies able to relatively quickly detect and quantify specific bacterial species in response to environmental changes, patient-specific recommendations and treatment for pathological conditions can be implemented as part of an improved treatment regimen. A more complete picture of pharmacological treatments and their interaction with the gut–bone axis [9] can lead to more optimized management of common orthopedic pathologies, including osteoporosis (Figure 2).

### 5.1. Pharmacological Gut Microbiota and Fracture Treatment: Known Interactions

While significant evidence regarding how alteration of the gut microbiota, mainly through the growing use of bioactive drugs and the cautious use of antibiotics, can impact human health exists, it only scratches the surface of the important effects that such drugs have on the gut microbiota and our vital homeostatic systems, especially those involved in bone health and healing after fracture. A growing number of literature reports raise the level of evidence and provide confidence that commonly prescribed drugs that significantly affect bone homeostasis may interact with other physiological systems, such as the gut microbiome, necessary for optimal bone function. Considering the growing body of evidence that links a healthy gut microbiota with bone homeostasis, the therapeutic benefits of commonly prescribed drugs and supplements, many of which are known to alter the gut microbiota [160,161], for osteopenia and osteoporosis should be reexamined. Common pharmaceutical interventions for osteoporosis, including active anti-osteoporotic agents (e.g., bisphosphonates, biologics), pain management (e.g., NSAIDs), and supplements (e.g., vitamin D and calcium), may synergistically improve or inadvertently harm a subset of patients based on the current status of their gut microbiota (i.e., healthy or unbalanced). Hence, considering a patient’s unique gut microbial composition and the apparent and created importance of the microbiota in bone health, future treatment approaches for osteoporosis must take an unprecedented, personalized approach. Unfortunately, gaps remain in the current understanding of the gut–bone axis that will make this challenging. More studies are needed to complete the picture and establish clear boundaries for a cause-and-effect relationship. Some of our most common osteoporosis treatments and their impact on the gut microbiota are explored below and are summarized in Table 2.

#### 5.1.1. Bisphosphonates

Although bisphosphonates are the first-line treatment for many patients with osteoporosis, few studies have been performed to determine any substantial link between the complex nature and function of the gut microbiome and bisphosphonate use. However, in a recent study, bisphosphonates were shown to have potential antiparasitic activity through interaction with the mevalonate (MVA) pathway, one of two primary pathways for synthesizing isoprenoids [162]. Until recently, it was thought that the MVA pathway was rarely used in bacteria; however, in a 2011 study on the genetic origins of the MVA pathway, several species of the phyla Bacteroidetes and Firmicutes, predominant phyla in the gut microbiome [163], were identified as having enzymes of the pathway [164]. In a similarly impactful 2017 study, Hayakawa et al. identified the MVA pathway and an MVA-like pathway in several additional bacterial species, some of which are opportunistic pathogens, leading them to hypothesize that the MVA pathway may provide an evolutionary advantage to these species [165]. Considering the presence and predominance of the MVA pathway bacterial species in the gut, the essential nature of the pathway for bacterial survival, and the documented inhibition of the pathway by bisphosphonates, it stands to reason that bisphosphonate use will alter the gut microbiota in some way. This hypothesis is indirectly supported by a recent report from Kalyan et al. that postulated that the long-term use of bisphosphonates could lead to gut microbiome-related immunosuppression associated with osteonecrosis of the jaw [166]. Therefore, although these medications classically have relatively benign side effects, the pharmacodynamic properties of the drugs, including a relatively long half-life, may have substantial unforeseen long-term effects on the body and their impact on the gut–bone axis needs to be critically evaluated.

#### 5.1.2. Monoclonal Antibodies

Denosumab, a humanized RANKL monoclonal antibody, is another medication used as a monotherapy or bisphosphonate-adjunct therapy for a subset of postmenopausal female patients with severe osteoporosis. In general, the safety profile of these anabolic medications is favorable. However, very little is known regarding any connection between the use of Denosumab on the human gut microbiome and how this interaction may affect overall bone health and healing. Nevertheless, there is indirect evidence based on a recent animal study by Khafipour et al. in mice with colitis, which demonstrated differing microbial diversity in mice treated with Denosumab [167]. Although the results have no direct bearing on bone homeostasis, the effects of Denosumab on bone health may either be enhanced or suppressed based on alterations in the gut microbiota. Alternatively, Romosozumab, an anti-sclerostin antibody, which temporarily stimulates bone formation, may also interact with the microbiota. Briefly, sclerostin is an osteocyte-produced paracrine-negative regulator (antagonist) of WNT signaling and both osteoblast and osteoclast activity at the bone surface [168]; thus, if it is inhibited with an antibody, WNT signaling is restored, osteoblastogenesis will be promoted, and osteoclasts will be inhibited [169,170,171]. Considering that anabolic WNT ligand expression (i.e., WNT10B) can be regulated by the SCFA butyrate produced by *Lactobacillus rhamnosus* through increased Treg cells [172], it stands to reason that Romosozumab may impact the gut microbiota through the WNT pathway. Such a possibility should be investigated considering the critical nature of butyrate in multiple aspects of bone metabolism. Ultimately, these potential treatment implications require urgent follow-up studies to investigate the direct connections between antibody-based osteoporosis treatments, changes in gut microbiota, and bone health [167].

#### 5.1.3. Hormonal Therapy

Estrogen promotes a healthy and diverse mix of commensal flora, enhancing T-regulatory cells’ function and suppressing inflammation [173]. Unfortunately, with the onset of menopause, sex steroid hormones are depleted, increasing: (1) the abundance of pathogenic bacteria; (2) the permeability of the small intestine, and (3) the production of osteoclastogenic factors such as IL17, RANKL, and TNF alpha [47]. Additionally, in a study by Fujiwara et al. in 2016, it was found that mice lacking RANKL in osteocytes were protected from increased osteoclast number and bone loss after ovariectomy. This is not entirely surprising; however, the same effect was seen when the α and β estrogen receptors were deleted on the B-cell surface, indicating that the increased bone marrow B-cell number, which is associated with decreased cancellous bone volume and estrogen deficiency, may be indirectly regulated by RANKL specifically produced by osteocytes [174]. Probiotics, specifically *L. reuteri*, reduced RANKL expression in an ovariectomized mouse, although the mechanism is not completely clear [175]. In a focused review of the overall effects of pharmaceuticals used to treat osteoporosis on the gut microbiota and their role in bone healing, estrogen therapy was linked to increased microbial diversity, decreased development of LPS-producing Gram-negative bacteria, changes in bacterial activity, tightening of gut permeability, and reduced inflammation caused by a high-fat diet. Based on a number of mouse and rat studies, Papageorgiou et al. proposed a feedback loop that ultimately influences the immune system. The microbially produced β-glucuronidase metabolizes estrogen, allowing it to be reabsorbed into circulation, interacting with its receptors and eliciting its biological actions [176]. It is this intimate connection between the gut microbiota and estrogen that leads to the suggestion that manipulation of the gut microbiota in conjunction with estrogen-based therapies may improve the overall inflammatory state and fortify bone mineral density [177], a suggestion that is supported by the spinal bone volume after ovariectomy in mice when treated with the probiotic, VSL#3 [178]. Papageorgiou asserts that hormones, including estrogens, significantly impact the efficacy of microbial metabolism, highlighting an integral but poorly understood relationship between endocrine therapy and the human gut microbiota [177].

#### 5.1.4. PTH-Related Analogs

PTH can induce both bone loss and bone formation based on the dosing regimen (intermittent or continuous) and the natural lifespan of an osteoblast, with intermittent dosing (daily administration) seeming to have a protective effect and continuous administration having no such effect [179]. Regardless of whether PTH promotes an anabolic or catabolic effect, the mechanisms of PTH are intimately tied to T-cell regulation and recruitment. In bone loss, generally segmented filamentous bacteria (SFB), a spore-forming, Gram-positive, commensal species, and several SFB-like bacteria are found. Previous work showed that SFB can induce differentiation into Th17 cells [180] and that PTH can induce Th17 cells in the small intestine but only in the presence of SFB. The PTH effect seems to result from the stimulation of TGFβ and TNF-producing T cells, which is mechanistically mediated by the presence of bacterial components (e.g., flagellin and lipopolysaccharide) [179]. Furthermore, Th17 cells are upregulated and recruited to the bone marrow, particularly in the presence of continuous PTH, where they secrete IL-17, ultimately causing bone destruction [181]. While no studies have directly investigated the relation between PTH-related analogs, such as Abaloparatide or Teriparatide, and the microbiota, Li and Yu have demonstrated that PTH anabolic-related processes are, in part, dependent on the microbiota through butyrate, a microbial-produced short-chain fatty acid [13,182]. More studies are needed to determine if PTH-related analogs, primarily used for their anti-osteoporotic effects, can be used to enhance the gut microbiota’s role in osteoporotic bone healing as part of a complex, multifactorial treatment regimen. It is also noteworthy to consider that for abaloparatide (more than teriparatide), nausea was the most commonly reported adverse event that led to study discontinuation [182] and that the emergence of gastrointestinal issues, in general, has been linked to alterations in microbial diversity [183]. Given the integral nature of the immune system and its hormonal regulation, more studies are needed to identify any direct link between PTH analogs, which may have differing and clinically relevant impacts on bone depending on the dosing regimen [123,125], and the composition of the gut microbiota.

#### 5.1.5. Non-Steroidal Anti-Inflammatory Drugs

Non-steroidal anti-inflammatory drugs (NSAIDs) are frequently used, in both prescription and over-the-counter forms (in 2014, it was reported that over 12% of Americans took an NSAID at least three times a week for three months [184]), to treat osteoporosis-related pain. In addition to their positive benefits on quality of life, NSAIDs may also positively affect bone healing; however, much of these effects are based on the dosing regimen and length of exposure [185]. Unfortunately, NSAIDs also have a well-documented history of adverse effects on bone healing, independent of the microbiota [185,186,187,188]. The negative impact on bone healing is based on the primary mechanism of action of NSAIDs, the adverse inhibition of COX-2, which has been suggested to be essential for bone fracture healing in a rat model [187], although this interpretation is not without controversy. Furthermore, there is evidence that the NSAID celecoxib also has an inhibitory effect on the Wnt/β-catenin signaling cascade, effectively blocking osteoblast maturation [189]. Although NSAIDs’ destructive effects on the gut through mucosal damage are relatively well known [190], their effects on the gut microbiome are just beginning to emerge. Notably, NSAIDs’ efficacy may also depend on metabolism or modification by the gut bacteria [191]. NSAIDs modify the gut microbiome, with different NSAIDs having slightly different effects [191,192], although such impacts largely have not been evaluated in the context of bone. Several preclinical studies in rodents have shown alterations in the microbiota upon administration of NSAIDs. Only two studies have considered NSAIDs’ impact in humans. In 2016, Bokulich et al. considered the use of celecoxib in postmenopausal, obese women and saw no effect on the microbiota composition detected in the feces; however, the study only considered a homogenous group of 10 women in a 10-day longitudinal study [193]. Furthermore, the Bokulich study seems to contradict a study from Thangamani et al. that considered the antimicrobial effects of celecoxib and concluded that it exhibited broad-spectrum activity against both Gram-positive and Gram-negative species, particularly when coupled with a membrane permeabilizing agent [194]. The study by Thangamani is supported by a recent in vitro study from Hernandez-Sanabria et al., who saw a reduction of 50% in butyrate produced in fecal microbiota cultures exposed to celecoxib [195]; considering the importance of butyrate in bone homeostasis, this could be a potentially very impactful finding if confirmed in vivo. Alternatively, Edogawa and colleagues considered the impact of indomethacin on both males and females and found extensive changes in both genders across multiple species [196]. In a rat study, indomethacin was shown to inhibit early-phase bone healing and formation [197]. Finally, the mechanistic effects of NSAIDs on the body as a whole create yet another possible way in which the treatment recommendations for osteoporosis may have multiple unforeseen effects, some of which will be mediated by alterations in the gut microbiota, that ultimately play a part in bone health.

**Table 2:** Hypothesized or known interactions between osteoporosis drug classes and gut microbiota.

Treatment	Gut Microbiota Interactions	References
Bisphosphonates	Long-term use is hypothesized to lead to gut microbiome-related immunosuppression via inhibition of the mevalonate (MVA) pathway.	[164,166]
Monoclonal Antibodies	Effects of Denosumab on bone health may either be enhanced or suppressed based on alterations in the gut microbiota.	[167,172]
	Romosozumab may impact the gut microbiota through the WNT pathway.	
Hormonal Therapy	Estrogen promotes a healthy and diverse mix of commensal flora, which improves T-regulatory cell function and suppresses inflammation, suggesting that manipulating the gut microbiota in conjunction with estrogen-based therapies may improve the overall inflammatory state and strengthen bone mineral density.	[177]
PTH-Related Analogs	More studies are needed to determine if PTH-related analogs can be used to enhance the gut microbiota’s role in osteoporotic bone healing.	[123,125]
Non-Steroidal Anti-Inflammatory Drugs (NSAIDs)	May mimic broad-spectrum antimicrobial properties.	[194,195]
	Inhibit some positive effects of microbiota through enzyme inhibition.	

## 6. Conclusions

A growing body of evidence suggests that the gut microbiota regulates bone metabolism and should be considered in the pathophysiology and treatment of osteoporosis. Current research reveals that bone homeostasis is linked to a healthy microbiome and that gut dysbiosis can exacerbate osteoclast activity and promote osteoporosis. Based on the literature, the human gut microbiota’s relationship with osteoblasts, osteoclasts, and receptor activator of nuclear factor-kappa-B ligand (RANKL) is critical in the modulation of osteoclastogenesis and osteoporosis. Additionally, micro-RNA, insulin-like growth factor 1, and immune system mediation are hypothesized as pathways of gut microbiome interaction with osteoclastogenesis and bone health in many studies. Although this is a complex relationship with several proposed mechanisms of modulation, addressing the microbiome in a treatment plan is not overly burdensome, yet it is predominately overlooked. No study has been done to report the exact number of medical practitioners who consider the microbiome in their treatment approach to osteoporosis or fracture healing. However, the connection, causal or associative, has been well defined and certainly warrants continued research, funding, and implementation in clinical medicine.

Drug–microbiome interactions are continually proving to be integral to treatment outcomes and can profoundly affect the gut–bone axis. Inquiries into the influence of oral bisphosphonates, the most common pharmacologic treatment for osteoporosis, on the human gut microbiota are limited in number and scope and should be expanded. The same applies to the other pharmaceutical treatment approaches considered in Table 1 and Section 5, emphasizing PTH analogs as they are increasingly prescribed and potentially beneficial to the microbiome. The ability to target the gut microbiota in the therapy of fracture and bone metabolic diseases such as osteoporosis opens new therapeutic possibilities and is a potential window of opportunity for exerting greater therapeutic control over the natural course of osteoporosis. The findings of this review support the recommendation for clinicians to pay the human gut microbiome its fair share of attention when considering the patient as a whole in the treatment of extra-gastrointestinal conditions such as osteoporosis. A clinician may recommend diet modification, probiotic-rich foods, or supplementation with probiotics or their metabolites, such as SCFAs, oligosaccharides, carbohydrates, and dietary fiber, to help to restore the gut flora balance in efforts to increase bone mineral density by promoting growth and restoring or maintaining the composition of intestinal bacteria.

Furthermore, the evidence supports the medicalization of the microbiota and the standardization of more conservative treatment approaches, including the regulation and approval of pre- and probiotics that are safe and efficacious. The Food and Drug Administration (FDA) has not yet approved any probiotic products to treat, mitigate, cure, or prevent certain diseases. However, since the FDA approved fecal microbiota transplantation (FMT) for the treatment of recurrent and refractory *Clostridium difficile* infection in 2013, FMT’s uses have expanded, not only in gastrointestinal problems but also in extra-gastrointestinal ailments [198]. Unfortunately, current pre- and probiotics are subject to the unregulated discretion of industrial nutraceutical labs, which produce probiotics in various ways, and whose ingredients and label claims are not tested or verified by the FDA. The effectiveness of a probiotic produced in these labs might vary from one brand to the next and even from batch to batch within the same brand. The above notwithstanding, and until a probiotic completes the drug development process and passes clinical trials, clinicians can and should still consider the microbiota in their treatment plans as medicine collectively navigates towards treating the whole patient and attempting to optimize all aspects of health while doing no harm, which veritably includes the microbiome.
